# MBEToolbox 2.0: An enhanced version of a MATLAB toolbox for Molecular Biology and Evolution

**Published:** 2007-02-06

**Authors:** James J. Cai, David K. Smith, Xuhua Xia, Kwok-yung Yuen

**Affiliations:** 1 Department of Biological Sciences, Stanford University, Stanford, CA, USA; 2 Department of Microbiology, University of Hong Kong, Hong Kong SAR, China; 3 Department of Biochemistry, University of Hong Kong, Hong Kong SAR, China; 4 Department of Biology, University of Ottawa, Ottawa, Ontario, Canada

**Keywords:** MBEToolbox, MATLAB, Molecular Evolution, Computer software

## Abstract

MBEToolbox is an extensible MATLAB-based software package for analysis of DNA and protein sequences. MBEToolbox version 2.0 includes enhanced functions for phylogenetic analyses by the maximum likelihood method. For example, it is capable of estimating the synonymous and nonsynonymous substitution rates using a novel or several known codon substitution models. MBEToolbox 2.0 introduces new functions for estimating site-specific evolutionary rates by using a maximum likelihood method or an empirical Bayesian method. It also incorporates several different methods for recombination detection. Multi-platform versions of the software are freely available at http://www.bioinformatics.org/mbetoolbox/.

## Introduction

MBEToolbox (from Molecular Biology and Evolution) is an integrated software package for sequence analysis under the MATLAB environment (Cai et al. 2005). The major new features in MBEToolbox version 2.0 include: (i) new functions for phylogenetic analyses by the maximum likelihood method; (ii) analysis of site-specific evolutionary rates; and (iii) algorithms to detect recombination. Implementing existing functions in the MATLAB framework greatly reduced the complexity of the original implementation and so allows users to enhance existing tools or add new functions much more easily than before.

The original version of MBEToolbox included sequence manipulation and statistics, evolutionary distance calculations, tree creation, a novel window analysis method and a graphical user interface. It differed from the MathWorks bioinformatics toolbox in that it provided a broader and deeper range of methods related to molecular evolution but did not cover areas such as microarray and mass spectrometry analyses. While there are some similarities between the two toolboxes in terms of basic sequence manipulation functions and the use of similar sequence input formats, version 2.0 of MBEToolbox widens the difference between the two packages for molecular evolutionary studies.

## Systems and Methods

MBEToolbox was developed and tested in MATLAB version 6.5 (R13) under Microsoft Windows. MBEToolbox 2.0 also runs on Sun-Solaris and Linux platforms with the same or a higher version of MATLAB installed. Minor effort might be required for other inter-platform migration, such as recompiling MEX files and obtaining appropriate versions of third party applications (e.g. ClustalW). Some functions require the installation of the MATLAB statistics and optimization toolboxes.

Speed is one of major concerns related to using interactive programming environments like MATLAB. Functions in MBEToolbox 2.0 have been analyzed using the MATLAB performance profiling tool (MathWorks Inc.) and the code was optimized where necessary. MATLAB is able to utilize code developed in other languages, such as C/C++ or FORTRAN, as its own dynamically linked subroutines (MEX files) which can be run from within MATLAB in the same way as MATLAB M-files or built-in functions. This is particularly useful if a MATLAB function cannot be fast enough, even after optimization. In these cases, ‘bottleneck’ functions have been written in C and converted into MEX files (which must be compiled for the specific platform the toolbox is to run on).

Much effort has been spent re-writing the code of MBEToolbox to increase the modularity of the functions and to facilitate their integration with other functions. This will allow users to add their own functions and enhancements more efficiently. A few minor bugs reported in version 1.0 have been fixed.

## Main New Features

### New Functions for Phylogenetic Analysis by Maximum Likelihood

MBEToolbox version 2.0 has extensively increased capabilities for phylogeny-based analyses using the maximum likelihood (ML) method.

Firstly, it now includes a function, dc_gy94, for estimation of synonymous (*dS*) and nonsynonymous (*dN* ) substitution rates using the GY94 codon substitution model (Goldman and Yang 1994). This function is fully-implemented in MATLAB. As a result, it is compact (less than 100 lines) and easier to be understood by users who may wish to extend it. To the best of our knowledge, this function is the only alternative implementation that can do this estimation, other than CODEML from the original author.

Secondly, inspired by (Zhang et al. 2006), we incorporated a novel codon substitution model-GY94m, a modification of the original GY94 model, into the toolbox. Compared to GY94, GY94m requires two parameters for transitions to allow substitution frequencies that can differ between the purines (A and G) and the pyrimidines (T and C). *κ_R_* and *κ_Y_* are used to differentiate the transition substitution frequencies of purines and pyrimidines, respectively. The substitution rate *q_ij_* from sense codon *i* to *j* to generate a transition probability matrix is as follows:

$$q_{ij,i\neq j}=\{\begin{array}{ll} 0, & \text{if }i\;\text{and }j\;\text{differ by more than}\;\text{one nucleotide difference}\\ \pi_{j}, & \text{if }i\;\text{and }j\;\text{differ by a synonymous transversion}\\ \kappa_{R}\pi_{j}, & \text{if }i\;\text{and }j\;\text{differ by}\;\text{a synonymous transtion between purines}\\ \kappa_{Y}\pi_{j}, & \text{if }i\;\text{and }j\;\text{differ}\;\text{by a synonymous transtion between pyrimidines}\\ \omega \pi_{j}, & \text{if }i\;\text{and }j\;\text{differ by a }\text{nonsynonymous transversion}\\ \omega \kappa_{R}\pi_{j}, & \text{if }i\;\text{and }j\;\text{differ by a nonsynonymous}\;\text{transtion between purines}\\ \omega \kappa_{Y}\pi_{j}, & \text{if }i\;\text{and }j\;\text{differ by}\;\text{a nonsynonymous transtion between pyrimidines} \end{array}$$

where π*_j_* is the stationary frequency of codon *j* and ωis the ratio of the nonsynonymous and synonymous substitution rates. If the transition rates between purines and between pyrimidines are set equal (*κ_R_* = *κ_Y_*), the model reduces to the GY94 model. With the GY94m model, we provide a new method for estimating *dS* and *dN* by the function dc_gy94m. This new method should improve estimation of *dS* and *dN* when unequal transition substitution rates become apparent, especially for sequences that are significantly diverged.

Finally, by combining different functions for ML phylogenetic analysis, one can use MBEToolbox to estimate tree topologies, branch lengths, and substitution parameters under a variety of nucleotide, protein or codon substitution models, and to perform likelihood ratio tests of positive selection or relaxed selective constraints, within a tree or between trees, based on the *dN/dS* ratios.

### Estimation of Site-specific Evolutionary Rate

Evolutionary rates in biological sequences are expected to vary among sites due to different selective constraints. Conserved sites may point to regions that are functionally and structurally important. MBEToolbox 2.0 estimates the site-specific relative evolutionary rate from a sequence alignment by either maximum likelihood (ML) or empirical Bayesian (EB) methods.

The ML method is considered to be a ‘state-of-the-art’ phylogenetic technique, allowing robust statistical testing of evolutionary hypotheses (Whelan et al. 2001). Estimation of site-specific rates by ML from both noncoding nucleotide and protein sequences have been described (Maidak et al. 1994, Pupko et al. 2002). These estimates are based on an alignment of sequences and a phylogeny. For coding nucleotide sequences, the estimation can be done by defining many rate categories in the gamma or discrete-rate models under the GY94 codon substitution model (Goldman and Yang 1994), by using software such as CODEML. Since MBEToolbox accommodates substitution models (noncoding, protein and codon substitution models) under a unified framework, the MBEToolbox 2.0 function rateofsit_ml can take an alignment of any type of sequences and estimate the site-specific evolutionary rates in terms of each nucleotide, amino-acid or codon site. The EB method function rateofsit_eb uses the method described in (Mayrose et al. 2004). It presupposes a prior distribution of evolutionary rates estimated by the ML method. Both ML and EB approaches have solid statistical foundations and are closely related, as they use the same models of evolution and operate within the same statistical framework. A demonstration example has been provided in the package.

### Recombination Detection

Recombination is one of the dominant forces shaping genomes and their associated phenotypes. The identification of recombination in aligned sequences is important for a better understanding of the impact of recombination on genome evolution (Posada et al. 2002). Different methods for detecting the presence of recombination have been established (See Posada et al. 2002 for review). These methods may be classified into five general categories: similarity, distance, phylogenetic, compatibility, and nucleotide substitution distribution methods. MBEToolbox 2.0 includes three recombination detection functions, plato, reticulate and stephen85, which belong to the phylogenetic, compatibility, and nucleotide substitution distribution methods, respectively. The function plato infers recombination when phylogenies from different parts of the genome result in discordant topologies or when orthologous genes from different species are clustered (Grassly and Holmes 1997). The reticulate function tests for incongruence in phylogenetic partitions on a site-by-site basis (Jakobsen and Easteal 1996). The function stephen85 examines sequences for a significant clustering of substitutions or their fit to an expected statistical distribution (Stephens 1985).

## Sliding Windows

MBEToolbox 2.0 can perform analyses by the sliding window method. Many statistics, like *dS* and *dN*, can be used in sliding window analyses. By taking advantage of function handles in MATLAB, we have greatly simplified the implementation of sliding window analysis. A function handle is a standard MATLAB data type which provides a means to call a function indirectly. All sliding window analyses share two common actions -sliding the window and plotting the calculated statistic. These two actions were implemented in one single function called slidingwin which takes any function handle as one of its inputs and calculates the corresponding statistic. The following example creates a function handle for the dnavariability function and assigns it to the variable fhandle:

≫fhandle = @dnavariability; 

The handle is passed to slidingwin in the same way one would pass any argument.

≫slidingwin(fhandle,…);

This passes the just created function handle of dnavariability to slidingwin, which then calculates the statistic by the function dnavariability, while moving the sequence window step by step and subsequently producing the plot. Users wishing to add new functions that require a sliding window analysis now only need to focus on their main analysis function and not re-implement a sliding window routine.

## Minor Improvements

Several sequence file input/output functions have been enhanced in MBEToolbox 2.0. It can output ‘pretty-printed’ (shaded) multiple alignments in HTML format, read different sequence alignment formats (PHYLIP, aligned FASTA and CLUSTAL) and convert (export) from one file format to another. Moreover, MBEToolbox 2.0 can retrieve multiple sequences from remote GenBank/GenPept databases and align them automatically. A user can provide a list of GenPept accession numbers and MBEToolbox 2.0 will retrieve the corresponding cDNA sequences and align them with respect to the coding frames, via one command.

## Availability

For academic users, MBEToolbox 2.0 and its source code are available free of charge from: http://www.bioinformatics.org/mbetoolbox/.
